# A combination of alveolar type 2–specific p38α activation with a high-fat diet increases inflammatory markers in mouse lungs

**DOI:** 10.1016/j.jbc.2025.108425

**Published:** 2025-03-19

**Authors:** C.K. Matthew Heng, Ilona Darlyuk-Saadon, Wupeng Liao, Manju P. Mohanam, Phyllis X.L. Gan, Nechama Gilad, Christabel C.M.Y. Chan, Inbar Plaschkes, W.S. Fred Wong, David Engelberg

**Affiliations:** 1 Department of Microbiology & Immunology, Yong Loo Lin School of Medicine, National University of Singapore, Singapore; 2 Singapore-HUJ Alliance for Research and Enterprise, Mechanisms of Liver Inflammatory Diseases Program, National University of Singapore, Singapore; 3 Department of Pharmacology, Yong Loo Lin School of Medicine, National University of Singapore, Singapore; 4 Department of Biological Chemistry, The Institute of Life Sciences, The Hebrew University of Jerusalem, Jerusalem, Israel; 5 Drug Discovery and Optimization Platform, Yong Loo Lin School of Medicine, National University Health System, Singapore; 6 Info-CORE, Bioinformatics unit of the I-CORE, The Hebrew University of Jerusalem, Jerusalem, Israel

**Keywords:** inflammation, lung, mitogen-activated protein kinase, obesity-associated lung disease, p38 MAPK, signal transduction, transgenic mice

## Abstract

Chronic respiratory diseases such as asthma and chronic obstructive pulmonary disease afflict millions of individuals globally and are significant sources of disease mortality. While the molecular mechanisms underlying such diseases are unclear, environmental and social factors, such as cigarette smoke and obesity, increase the risk of disease development. Yet, not all smokers or obese individuals will develop chronic respiratory diseases. The mitogen-activated protein kinase p38α is abnormally active in such maladies, but its contribution, if any, to disease etiology is unknown. To assess whether p38α activation *per se* in the lung could impose disease symptoms, we generated a transgenic mouse model allowing controllable expression of an intrinsically active variant, p38α^D176A+F327S^, specifically in lung alveolar type 2 pneumocytes. Sustained expression of p38α^D176A+F327S^ did not appear to induce obvious pathological outcomes or to exacerbate inflammatory outcomes in mice challenged with common respiratory disease triggers. However, mice expressing p38α^D176A+F327S^ in alveolar type 2 cells and fed with a high-fat diet exhibited increased numbers of airway eosinophils and lymphocytes, upregulated levels of proinflammatory cytokines and chemokines including interleukin-1**β** and eotaxin, as well as a reduction in levels of leptin and adiponectin within the lung. Neither high-fat diet nor p38α^D176A+F327S^ alone induced such outcomes. Perhaps in obese individuals with associated respiratory diseases, elevated p38α activity which happens to occur is the factor that promotes their development.

Chronic respiratory diseases refer to a group of heterogenous conditions characterized by prolonged inflammation of the lungs and airways. These include diseases such as asthma, chronic obstructive pulmonary disease (COPD), and idiopathic pulmonary fibrosis (IPF)—illnesses that collectively afflict millions of individuals globally. These diseases are currently incurable, posing a significant burden to society (1, 2, 3).

The causes of chronic respiratory diseases are unclear. Unlike cancers, for example, which can be caused by a single component, no such individual mutation or gene amplification has been identified as a cause of such conditions. Chronic respiratory diseases, like other chronic inflammatory diseases (CIDs), are believed to be multifactorial in nature and involve the integration of various environmental, genetic, and social factors (4, 5, 6, 7). Many triggers and risk factors for such conditions have been identified, yet no definitive cause has been reported thus far. For example, obesity and cigarette smoke are well-established risk factors for asthma and COPD, respectively. However, not all obese individuals or smokers will develop such conditions (8, 9).

The lung is a highly complex tissue consisting of numerous cell types, many of which drive various pathological mechanisms in chronic respiratory diseases (5, 10). For example, resident alveolar macrophages (AMϕ) are the majority population within the alveolar airspace and considered to be “first responders” of the lung. Their known contributions to chronic respiratory diseases include the production of proinflammatory signaling molecules, fibrotic build-up, and dysfunctional airway clearance (11).

Another notable pulmonary cell subpopulation is the alveolar type 2 (AT2) pneumocyte. AT2 cells are progenitor cells of the alveolar epithelium, capable of differentiating into alveolar type 1 (AT1) cells for gaseous exchange following insult or injury. Simultaneously, they regulate alveolar homeostasis *via* cytokines, lung surfactants, and extracellular vesicles, among others (12). Research on the alveolar epithelium in chronic respiratory diseases has largely focused on AT2 cells, implicating them in hallmarks of pathology such as inflammatory signaling, accelerated ageing, as well as epithelial damage via apoptosis or fibrotic buildup (13, 14).

Chronic respiratory disease models usually involve exposure to disease triggers such as lipopolysaccharides (LPSs) or house dust mite (HDM) extract in regular or obese mice, as well as genetic manipulation such as alpha-1-antitrypsin knockouts (15, 16, 17, 18). Due to the complexity of the lung and the diseases themselves, there is no available model that can recapitulate such conditions in their entirety.

Although the abnormal activity of a single enzyme or pathway is not known to be sufficient of the development of CIDs, a common denominator shared by possibly all CIDs at the biochemical level is elevated activity of the mitogen-activated protein kinase (MAPK) p38α (19, 20). Increased p38α activity is associated with hallmarks of chronic respiratory disease such as airway inflammation and remodeling (19), and its inhibition has demonstrated promise in preclinical animal models and early-stage clinical trials, making it an attractive target for therapeutic modulation (21, 22). p38α activity is also elevated in the aged lung (23) and many chronic respiratory diseases are age-related. While no p38α inhibitors have made it to the clinic thus far (24), the observations following its inhibition suggest it is central to chronic respiratory disease pathology. Yet, attempts to understand if p38α activation is a driver or perhaps more of a supporting actor in such maladies have been hindered by the lack of a method allowing for its activation *per se* in the lung.

To achieve p38α activation *per se* in a tissue-specific manner, we established a transgenic mouse model allowing for controlled expression of an active variant of p38α, p38α^D176A+F327S^ (25, 26, 27, 28; the model is described in references (27) and (28)). Among several intrinsically active p38α molecules described previously (26, 29), p38α^D176A+F327S^ was chosen as it exhibited the highest spontaneous activity *in vitro*. Furthermore, we had observed in prior work that expression of p38α^D176A+F327S^ in all mouse tissues led to physiological changes in the lungs (28). Given the association of p38α with chronic lung diseases, we restricted p38α^D176A+F327S^ expression to AT2 cells in the mouse lung for the current study. We report that induction of p38α^D176A+F327S^ in AT2 cells caused changes in the AMϕ population, which contained an increased proportion of arginase-1^+^ (Arg1^+^) M2-like AMϕ. Elevation of surfactant-associated protein D (SP-D) production was also noted. Yet, no significant respiratory disease hallmarks were observed. Challenging mice expressing AT2-specific p38α^D176A+F327S^ with intratracheal LPS or HDM extract also did not exacerbate disease symptoms induced by these agents. However, when mice expressing p38α^D176A+F327S^ were provided with a high-fat diet (HFD), we observed increased numbers of eosinophils and lymphocytes within the lung along with the upregulation of various proinflammatory cytokines and chemokines. There was also a notable decrease in the levels of leptin and adiponectin within the lung, with adiponectin in particular considered to be anti-inflammatory in nature.

## Results

### Establishing a mouse model for the inducible lung-specific expression of p38**α**^D176A+F327S^

To assess whether p38α could cause symptoms of respiratory inflammation, either alone or in combination with other triggers, we sought to establish an experimental system for lung-specific p38α activation. We used the “carrier” mouse harboring complementary DNA (cDNA) encoding for p38α^D176A+F327S^ as described in references (27) and (28). A schematic depiction of the gene cassette used is included in Fig. S1. As shown in Fig. S1, the use of the Cre-lox and TetOn systems ensures that p38α^D176A+F327S^ can only be expressed in cells expressing Cre recombinase (Cre) and in the presence of a tetracycline such as doxycycline (Dox). By crossing these “carrier” mice with a lung-specific Cre “driver” mouse, their progeny should express p38α^D176A+F327S^ in the lungs when provided with a Dox-supplemented diet.

As no mouse constitutively expressing Cre in a lung-specific manner was available, we generated a mouse strain capable of constitutive Cre expression in AT2 cells. The Cre gene was inserted as an internal ribosome entry site-Cre-polyA sequence into the exon 6 region downstream of the translation termination codon of *Sftpc*. *Sftpc* is transcribed under an AT2 cell–specific promoter (30). The internal ribosome entry site approach ensures constitutive expression of Cre in a lung-specific manner while preserving the endogenous *Sftpc* gene. Mice which exhibited lung-specific constitutive Cre expression were termed “SFTPC-Cre” mice. SFTPC-Cre mice were then crossed with C57BL/6J mice for two generations before use.

The resulting F_3_ generation SFTPC-Cre mice were bred with the p38α^D176A+F327S^ “carrier” mice. Progeny harboring both transgene constructs, the SFTPC-Cre and the p38α^D176A+F327S^ expression cassette, were selected. In theory, these mice would constitutively express Cre and rtTA in an AT2-specific manner. When dox is provided to these mice, the resulting dox–rtTA complex induces transcription of p38α^D176A+F327S^ via a Tet-On promoter. In this manner, expression of p38α^D176A+F327S^ would be restricted to lung AT2 cells and induced only when dox is available.

**Figure 1:**
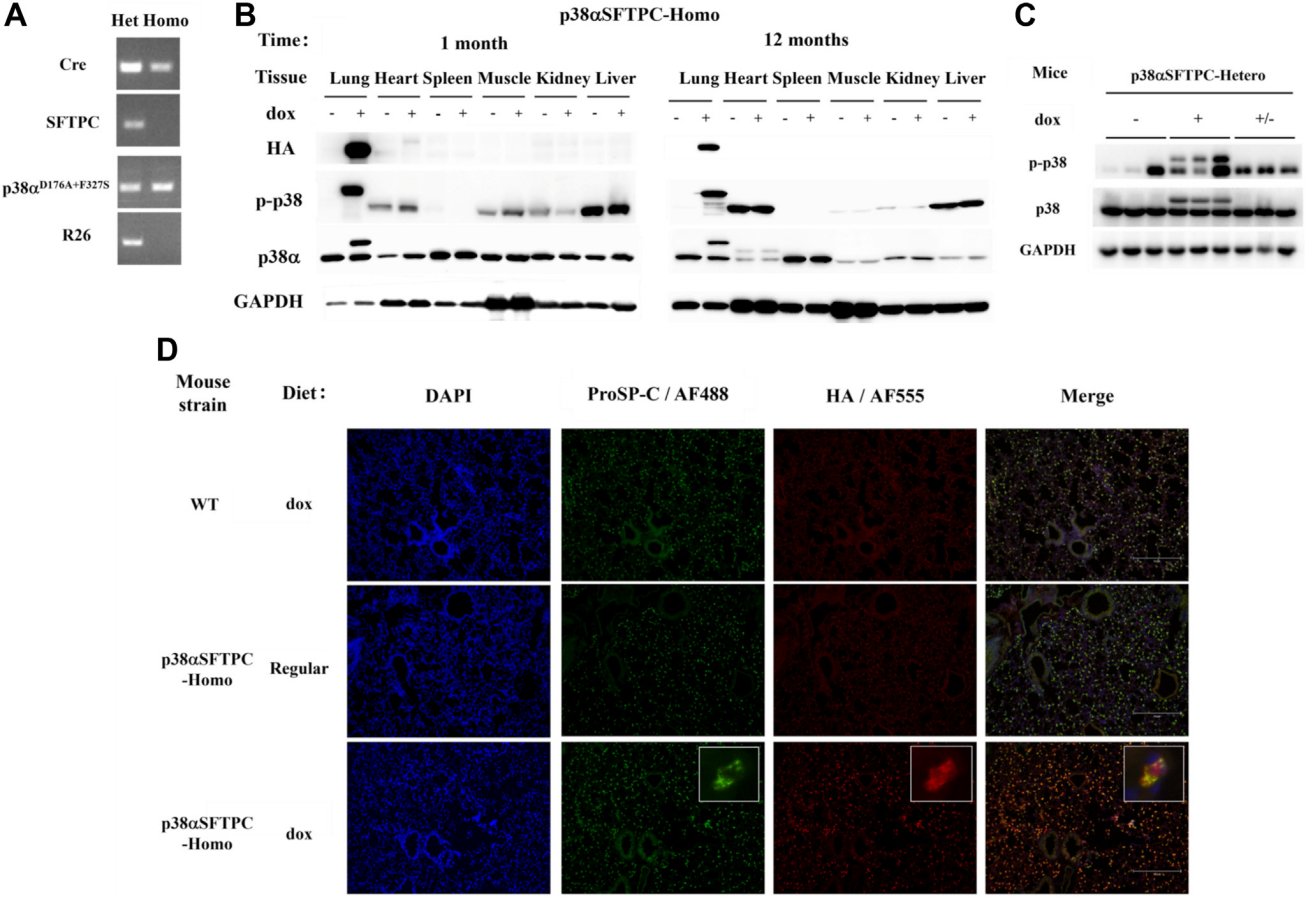
**Establishment of a mouse model, termed p38αSFTPC mice, allowing for induced expression of p38α^D176A+F327S^ exclusively in lung AT2 pneumocytes.***A*, PCR products for identifying the genotype of inbred progeny. “Hetero” (*left lane*) is heterozygous for both SFTPC-Cre and p38α^D176A+F327S^, while “Homo” (*right lane*) is homozygous for both SFTPC-Cre and p38α^D176A+F327S^. Representative gels are shown. *B*, Western blot analysis of lysates prepared from the indicated tissues, removed from p38αSFTPC mice provided with a regular diet (−) or Dox-supplemented diet (+) for 1 (*left panel*) or 12 (*right panel*) months. Blots were probed with the indicated antibodies. *C*, Western blot analysis of lung lysates prepared from three groups of p38αSFTPC mice. 1. Mice provided with a regular diet for 2 weeks (−), 2. Mice provided with a Dox-supplemented diet for 2 weeks (+), and 3. Mice provided with a Dox-supplemented diet for 1 week and then “reversed” to a regular diet for 1 week (+/−). Blots were probed with the indicated antibodies. *D*, paraffin-embedded lung sections were obtained from WT and p38αSFTPC-homo mice provided with the indicated diet for 2 weeks. Sections were immunostained with antibodies against proSP-C (AT2 marker) or HA (HA-p38α^D176A+F327S^). Nuclei were counterstained with DAPI. An inset in the images of the *bottom row* shows the colocalization of AF488-ProSPC and AF555-HA. Three images per section were taken from each mouse at 100x magnification and representative images are shown (n = 3). *B* and *C*, representative blots are used (n = 3–6 for each time point). AT2, alveolar type 2; DAPI, 4',6'-diamino-2-phenylindole; ProSP-C, prosurfactant protein C; Dox, doxycycline.

Progeny from this breeding strategy, which are heterozygous for both transgene constructs, were termed “p38αSFTPC-heterozygous” (p38αSFTPC-hetero). p38αSFTPC-hetero mice were further inbred to obtain progeny possessing two copies of the p38α^D176A+F327S^ allele and at least one copy of the SFTPC-Cre allele. These mice were termed “p38αSFTPC-homozygous” (p38αSFTPC-homo) mice (Fig. 1*A*).

### Expression of p38**α**^D176A+F327S^ in the p38**α**SFTPC mouse model is restricted to the lungs and dependent on provision of Dox

To confirm lung-specific expression of p38α^D176A+F327S^ and its dependence on the provision of Dox, p38αSFTPC-hetero and p38αSFTPC-homo mice were provided with a Dox-supplemented diet for time periods of up to 12 months. Following this, various tissues were collected. Western blot analysis revealed that expression of p38α^D176A+F327S^ is restricted to the lungs of p38αSFTPC mice and dependent on the presence of dox. No “leaky” expression was detected in other tissues or in p38αSFTPC mice provided with regular diet (Fig. 1*B*). Importantly, the p38α active variant, expressed in the lung, retained its ability to spontaneously autophosphorylate upon expression and remained active/phosphorylated for at least 12 months (Fig. 1*B*). When p38αSFTPC mice had their diet “reversed,” that is, reverted from a Dox-supplemented to regular diet, p38α^D176A+F327S^ expression was abolished within 24 h of providing the mice with a regular diet (Fig. 1*C*).

To confirm AT2-specific expression of p38α^D176A+F327S^, we performed immunofluorescent staining of formalin-fixed paraffin-embedded lung tissue sections. Expression of the haemmagglutinin (HA)-tag was found to colocalize with prosurfactant protein C (ProSP-C), an established marker of AT2 cells, only in p38αSFTPC-homo mice provided with a Dox-supplemented diet (Fig. 1*D*). In AT1 cells, identifiable by their morphology as long and thin cells which surround the alveolar airspaces, no HA-tag expression was detected (Fig. 1*D*), denoting a lack of p38α^D176A+F327S^ in those cells. Thus, p38α^D176A+F327S^ is expressed specifically in AT2 cells only following the provision of a Dox-supplemented diet.

### p38**α**SFTPC-homo mice provided with a Dox-supplemented diet display no obvious airway inflammation following sustained activation of p38**α**

**Figure 2:**
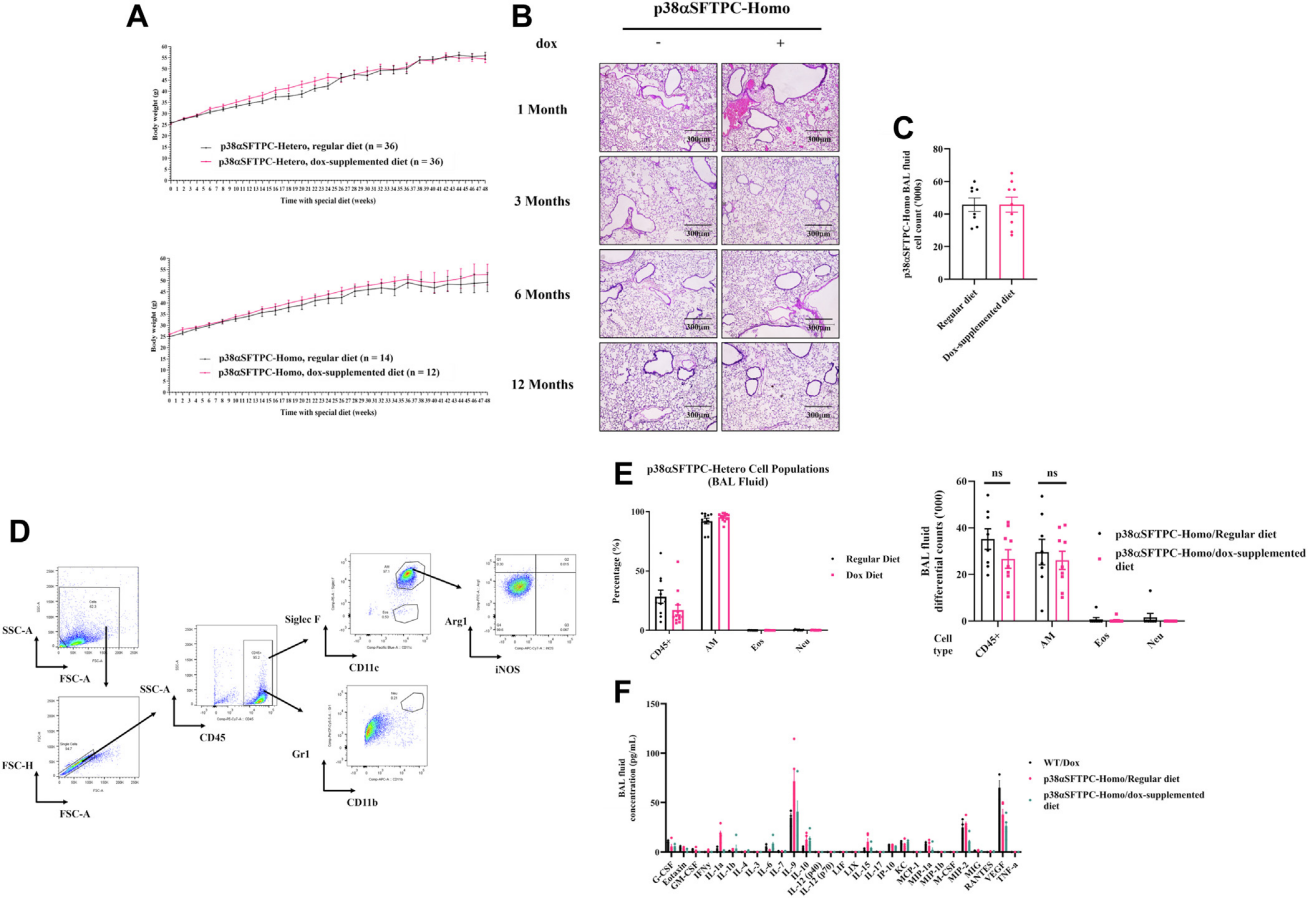
**Expression of p38α^D176A+F327S^ did not result in noticeable adverse outcomes or pathological changes in the lungs of p38αSFTPC mice.***A*, p38αSFTPC-hetero (*top panel*) and p38αSFTPC-homo mice (*bottom panel*) were provided with either a Dox-supplemented or a regular diet and weighed every 2 weeks for up to 12 months. The average weight of each group over the 12 months is presented (n = 12–36). *B*, lung sections obtained from p38αSFTPC-homo mice provided with a Dox-supplemented or a regular diet for 1, 3, 6, and 12 months were H&E stained. Sections were imaged at 100x magnification and representative images are shown (n = 3). *C*, counts of cells obtained from BAL fluid of p38αSFTPC-homo mice provided with a Dox-supplemented or regular diet for 1, 3, and 6 months (n = 8). *D*, FACS gating strategy to identify immune cell subsets in the BAL fluid. Immune cells are defined as CD45^+^. After gating on immune cells, AMϕ are defined as CD45^+^CD11c^+^Siglec F^+^, while eosinophils are defined as CD45^+^CD11c^-^Siglec F^+^. Neutrophils are defined as CD45^+^CD11b^+^Gr-1^+^. After gating on AMϕ, M1-like AMϕ are defined as iNOS^+^, while M2-like AMϕ are defined as Arg-1^+^. *E*, BAL fluid cell composition from p38αSFTPC-hetero mice (*left panel*; n = 9) and differential counts of BAL fluid cells from p38αSFTPC-homo mice (*right panel*; n = 7–8) fed with a Dox-supplemented or regular diet for up to 6 months. The total percentage of CD45+ immune cells is represented in the *leftmost column*, which is further broken down into AM, eosinophil (Eos), and neutrophil (Neu) subpopulations. *F*, level of cytokines and chemokines in BAL fluid from p38αSFTPC-homo mice provided with a Dox-supplemented or regular diet for 2 weeks, and from WT mice provided with a Dox-supplemented diet (n = 3–4). AMϕ, alveolar macrophages; Arg1^+^, arginase-1^+^; BAL, bronchoalveolar lavage; Dox, doxycycline; FACS, fluorescence-activated cell sorting; iNOS, inducible nitric oxide synthase.

p38αSFTPC-hetero and p38αSFTPC-homo mice expressing p38α^D176A+F327S^ did not exhibit any outward signs of ill health even after 12 months. They exhibited similar weight gain to that of control mice and appeared healthy (Fig. 2*A*). H&E staining of lung sections revealed no significant structural issues or cellular infiltration into the alveolar airspaces in p38αSFTPC-homo mice that had been provided with a Dox-supplemented diet for up to 12 months (Fig. 2*B*). This was further confirmed with bronchoalveolar lavage (BAL) fluid cell counts, revealing no significant differences in the average number of cells within the airways of p38αSFTPC mice regardless of whether they were given a Dox-supplemented or regular diet (Fig. 2*C*).

Overall, there were no signs to suggest that chronic activation of p38α in the alveolar epithelium was leading to the development of obvious inflammatory outcomes in the lung. We used fluorescence-activated cell sorting (FACS) to identify CD45^+^ immune cells before going deeper to identify resident AMϕ (CD45^+^CD11c^+^SiglecF^+^), eosinophils (CD45^+^CD11c^-^SiglecF^+^), and neutrophils (CD45^+^CD11b^+^Gr1^+^). Resident AMϕ were further gated on inducible nitric oxide synthase and Arg1 to delineate M1-like and M2-like polarization states, respectively. Our gating strategy is illustrated in Figure 2*D*. This analysis revealed that AMϕ dominate the airways of p38αSFTPC mice (>90%) regardless of whether they had received a Dox-supplemented or regular diet, with negligible infiltration of other immune cells being detected (Fig. 2*E*). A Luminex assay measuring 29 cytokines and chemokines was done using BAL fluid with no significant changes observed in the lungs of p38αSFTPC-homo mice provided with a Dox-supplemented diet for 2 weeks (Fig. 2*F*). These observations stand in contrast to those from mouse models expressing p38α^D176A+F327S^ in other organs. When expressed across the whole body, p38α^D176A+F327S^ caused anemia and premature mortality (28). When expressed exclusively in the liver, it triggered symptoms of fatty liver (27). Finally, when expressed in skeletal muscle, it induced signs of regeneration, suggesting recovery from damage (Gilad *et al.*, unpublished data).

### Minimal effect of p38**α**^D176A+F327S^ expression in AT2 cells on the lung’s transcriptome

Accordingly with the lack of an apparent phenotype, the effect of p38α^D176A+F327S^ expression on the lung transcriptome, which was obtained via RNA-seq, was mild. In two independent experiments, relatively few genes were differentially expressed, with *Nrip3* and *Sprr1a* among the most significantly upregulated (Fig. S2, *A* and *B*). The surfactant-associated proteins *Sftpa1* and *Sftpd* were mildly upregulated following p38α^D176A+F327S^ expression, although there was a surprising downregulation of *Sftpc* in all p38αSFTPC mice regardless of p38α^D176A+F327S^ expression (Fig. S2*B*). Overall, most of the lung mRNA repertoire appeared similar regardless of p38α^D176A+F327S^ expression (provided in Supporting information, Datasets S1 and S2).

RNA-seq also revealed no downregulation of MAPK kinase 6 (MKK6) mRNA (Files S1 and S2), which we previously demonstrated to be reduced following p38α^D176A+F327S^ expression in skeletal muscle (Gilad *et al.*, unpublished data). Likewise, no significant differences were observed in the expression levels of signature genes for the various alveolar epithelial cell subpopulations such as AT1 cells or damage-associated transient progenitors (31).

### p38**α**^D176A+F327S^ expression caused the elevation of Arg1 levels in AMϕ

**Figure 3:**
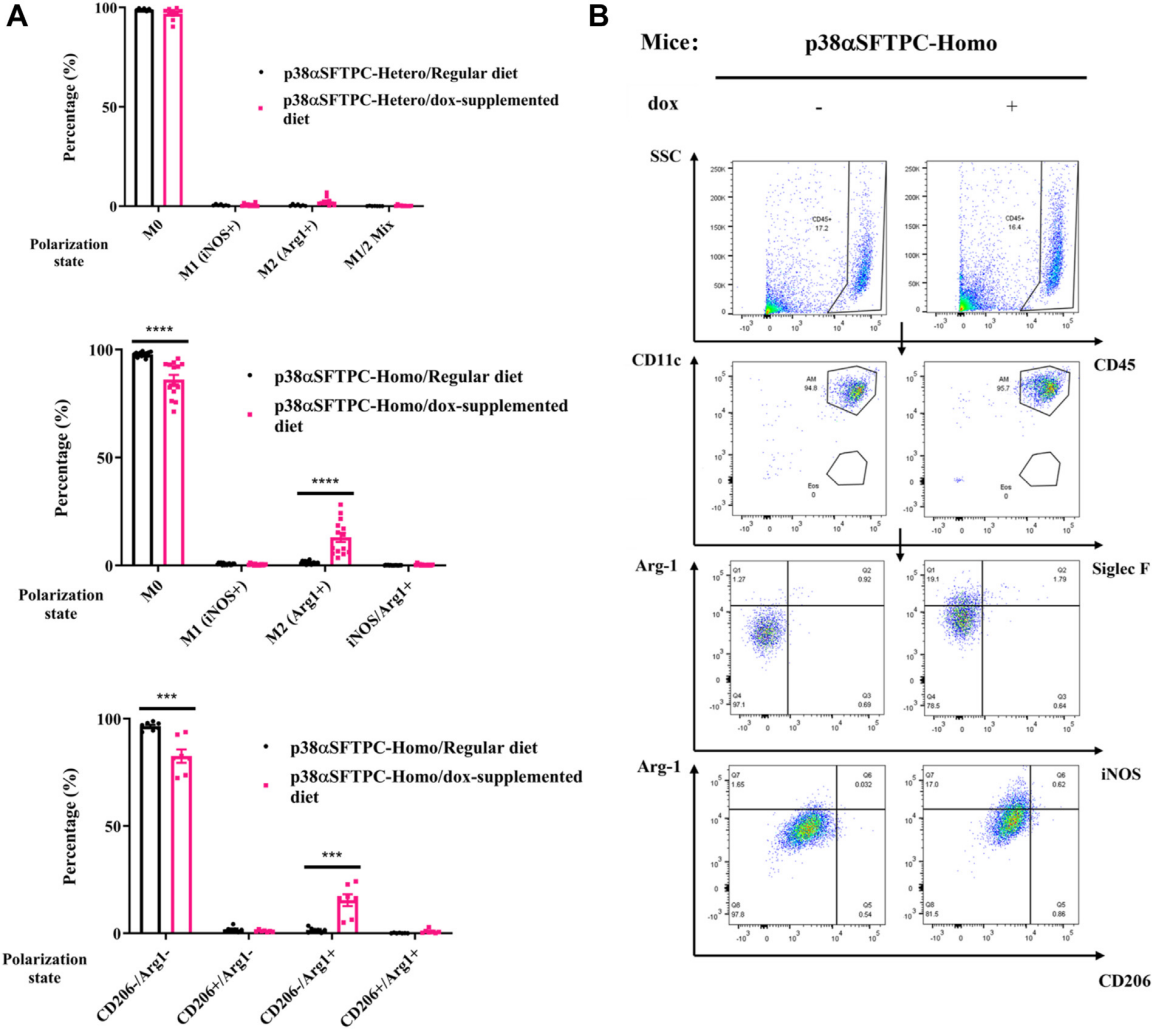
**AT2-specific expression of p38α^D176A+F327S^ caused an upregulation of Arg1^+^ M2-like AMϕ in p38αSFTPC-homo mice.***A*, percentage of BAL fluid AMϕ expressing the polarization markers iNOS and Arg1. Shown are analyses of BAL fluid taken from p38αSFTPC-hetero (*upper panel*) and p38αSFTPC-homo (*middle panel*) mice provided with a Dox-supplemented or regular diet for timepoints of up to 12 months. The *bottom panel* shows the percentage of BAL fluid AMϕ expressing Arg1 and CD206 from p38αSFTPC-homo mice provided with a Dox-supplemented or regular diet for 9 to 12 months *B*, the illustrated gating strategy was applied to monitor M2-like AMϕ polarization based on Arg-1, iNOS, and CD206 expression. Representative plots are shown. *A* and *B*, (n = 7–15). AMϕ, alveolar macrophage; Arg1, arginase-; BAL, bronchoalveolar lavage; Dox, doxycycline; iNOS, inducible nitric oxide synthase.

While AT2-specific p38α^D176A+F327S^ did not affect the number or composition of airway immune cells (Fig. 2, *C*–*E*), flow cytometry revealed an effect on AMϕ maturation (Fig. 3). In lungs expressing active p38α, a large percentage (19.1%) of AMϕ expressed upregulated levels of Arg1, a marker suggesting M2-like polarization (32), compared to 1.27% of AMϕ in p38αSFTPC-homo mice not expressing p38α^D176A+F327S^ (Fig. 3*A*; top/middle panels; Fig. 3*B*). However, the Arg1^+^ AMϕ in p38αSFTPC-homo mice displayed no differences in the level of CD206, another known M2 macrophage marker (33) (Fig. 3*A*, bottom panel; Fig. 3*B*, *bottom row*). A plausible explanation is that upon activation of p38α, AT2 cells secrete a currently unidentified factor which can induce partial M2-like polarization in AMϕ. Notably, M2-like maturation was also observed in the lungs of mice expressing p38α^D176A+F327S^ across the entire organism (28). Intriguingly, although p38α activation is expected to promote inflammation, the M2-like state is more commonly associated with the repair and resolution of inflammation (34). Perhaps the activation of p38α might instead be beneficial to patients under certain pathological conditions (See Discussion; Ref. 52).

### AT2-specific expression of p38**α**^D176A+F327S^ caused upregulation of surfactant-associated proteins

**Figure 4:**
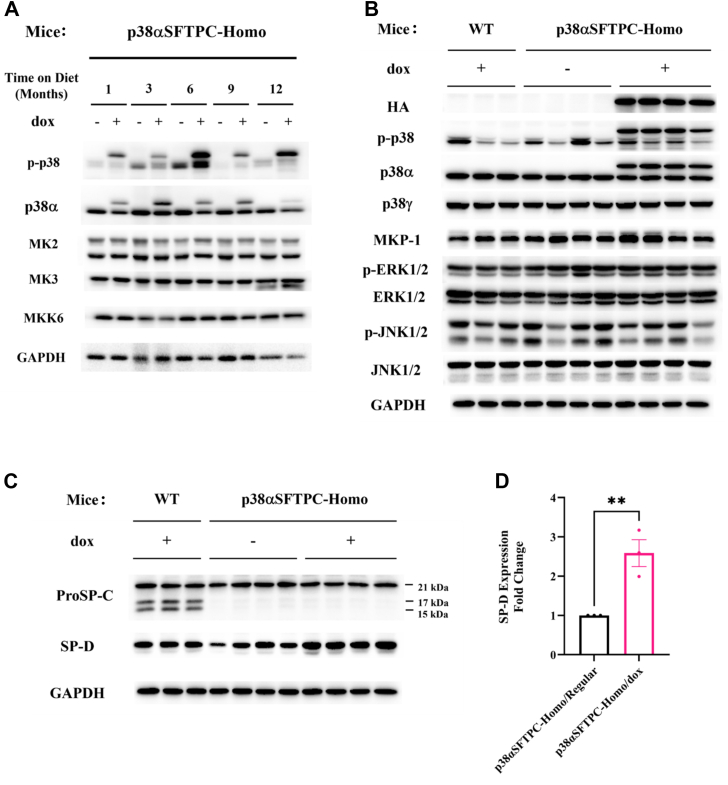
**AT2-specific expression of p38α^D176A+F327S^ did not affect steady-state levels of p38α pathway components in the lung.***A*, Western blot analysis with the indicated antibodies of whole lung lysates prepared from p38αSFTPC-homo mice provided with a Dox-supplemented or regular diet for 1, 3, 6, 9, and 12 months *B*, Western blot analysis with the indicated antibodies of whole lung lysates prepared from p38αSFTPC-homo or WT mice provided with the indicated diet for 2 weeks. *C*, Western blot analysis with the indicated antibodies of whole lung lysates prepared from p38αSFTPC-homo or WT mice provided with the indicated diet for 2 weeks. The molecular weight of ProSP-C is ∼21 kDa, while processing intermediates are ∼17 kDa and ∼15 kDa. The GAPDH image used is the same as in (*B*) as it was obtained with the same samples within the same assay. The separation between (*B*) and (*C*) is simply to better organize our findings. *D*, quantification of SP-D levels across three time points of 2 weeks, 3 months, and 12 months *A*–*C*, representative blots are shown (n = 3–4 for each time point). AT2, alveolar type 2; SP-D, surfactant-associated protein D; ProSP-C, prosurfactant protein C.

As p38α^D176A+F327S^ was not appearing to impose any significant phenotype on the lungs or markedly alter gene expression signatures by itself, we wondered about its effects at the biochemical level. Previous studies with similar mouse models as well as in cell culture systems showed that chronic p38α activity downregulates the expression levels of its substrates MK2 and MK3, its activator MKK6, as well as endogenous p38α proteins (27, 28; Gilad *et al.*, unpublished data). In contrast to its effect in other tissues, p38α^D176A+F327S^ expression did not downregulate MK2, MK3, MKK6, or endogenous p38α at the whole lung level in p38αSFTPC-homo mice (Fig. 4*A*). Similarly, levels of other MAPKs and the MAPK phosphatase 1 were not affected by p38α^D176A+F327S^ (Fig. 4*B*). Finally, levels of p38γ, a p38 isoform implicated in IPF (35), were also similar in mice regardless of p38α^D176A+F327S^ expression (Fig. 4*B*).

Nonetheless, provision of a Dox-supplemented diet to p38αSFTPC-homo mice induced upregulation (∼2.5-fold) in the expression level of SP-D at time points of up to 12 months (Fig. 4, *C* and *D*). Notably, upregulated SP-D is noted in conditions such as COPD and allergic asthma, where it is assumed to be part of the lung’s response to ongoing injury and inflammation (36).

### Expression of p38**α**^D176A+F327S^ did not result in increased disease severity when combined with respiratory disease models

**Figure 5:**
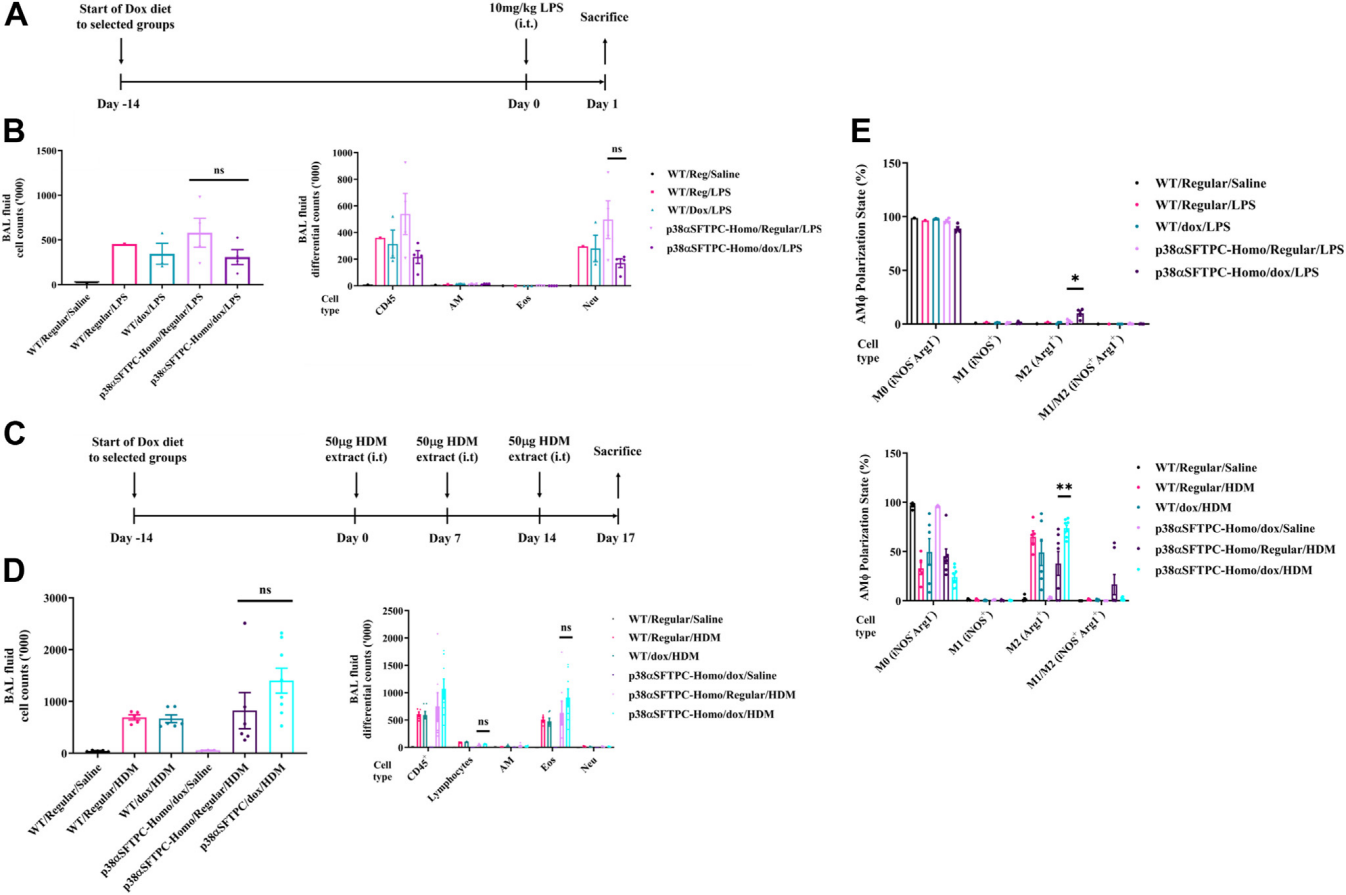
**AT2-specific p38α^D176A+F327S^ expression did not exacerbate inflammatory outcomes in the lungs of mice challenged with intratracheal LPS or HDM extract.***A*, timeline for the 24 h LPS-induced acute lung inflammation model. *B*, BAL fluid cell counts (*left graph*) and differential counts (*right graph*) after challenge with intratracheal 10 mg/kg LPS. *C*, timeline for the HDM-induced allergic asthma model. *D*, BAL fluid cell counts (*left graph*) and differential counts (*right graph*) after challenge with intratracheal 50 μg HDM extract. *E*, percentage of AMϕ expressing the polarization markers iNOS and Arg1 following challenge with LPS (*upper panel*) or HDM extract (*lower panel*). *A*, *B*, and *E*, “WT/Reg/saline” and “WT/Reg/LPS” were used to determine the basal response level of the models (n = 1). *A*–*E*, all mice were kept on their respective Dox-supplemented/regular diet throughout the duration of the disease model (n = 3–8). AMϕ, alveolar macrophage; Arg1^+^, arginase-1^+^; AT2, alveolar type 2; BAL, bronchoalveolar lavage; Dox, doxycycline; HDM, house dust mite; HFD, high-fat diet; iNOS, inducible nitric oxide synthase; LPS, lipopolysaccharide.

Unlike p38α^D176A+F327S^ expression in liver or skeletal muscle, where it alone was sufficient to cause pathological symptoms, its expression *per se* in the lung was insufficient to cause apparent lung pathology. To check if it could exacerbate the effect(s) of other respiratory disease triggers, we combined p38α^D176A+F327S^ expression with a 24 h model of LPS-induced acute lung inflammation (Fig. 5*A*) and a 2-week HDM-induced allergic asthma model (Fig. 5*C*). As expected, LPS exposure induced significant neutrophil infiltration. However, the effects of LPS were similar regardless of p38α^D176A+F327S^ expression in the lungs of p38αSFTPC-homo mice (Fig. 5*B*).

Similarly in the 2-week HDM-induced allergic asthma model, p38α^D176A+F327S^ expression did not exacerbate pathological outcomes as determined by the number of infiltrating lymphocytes and eosinophils (Fig. 5*D*). With regards to AMϕ polarization in both disease models, Arg1^+^ M2-like AMϕ were upregulated in mice provided with a Dox-supplemented diet (Fig. 5*E*). Interestingly, M2-like AMϕ are known to be upregulated in allergic asthma (37) and this appears to be amplified by p38α^D176A+F327S^ expression. About 37.91% of AMϕ in p38αSFTPC-homo mice given a regular diet and challenged with HDM were Arg1^+^, compared to ∼73.81% in p38αSFTPC-homo mice provided with a Dox-supplemented diet and challenged with HDM (Fig. 5*E*; bottom panel).

### p38**α**^D176A+F327S^ expression combined with an HFD induces infiltration of eosinophils and lymphocytes into the lung

**Figure 6:**
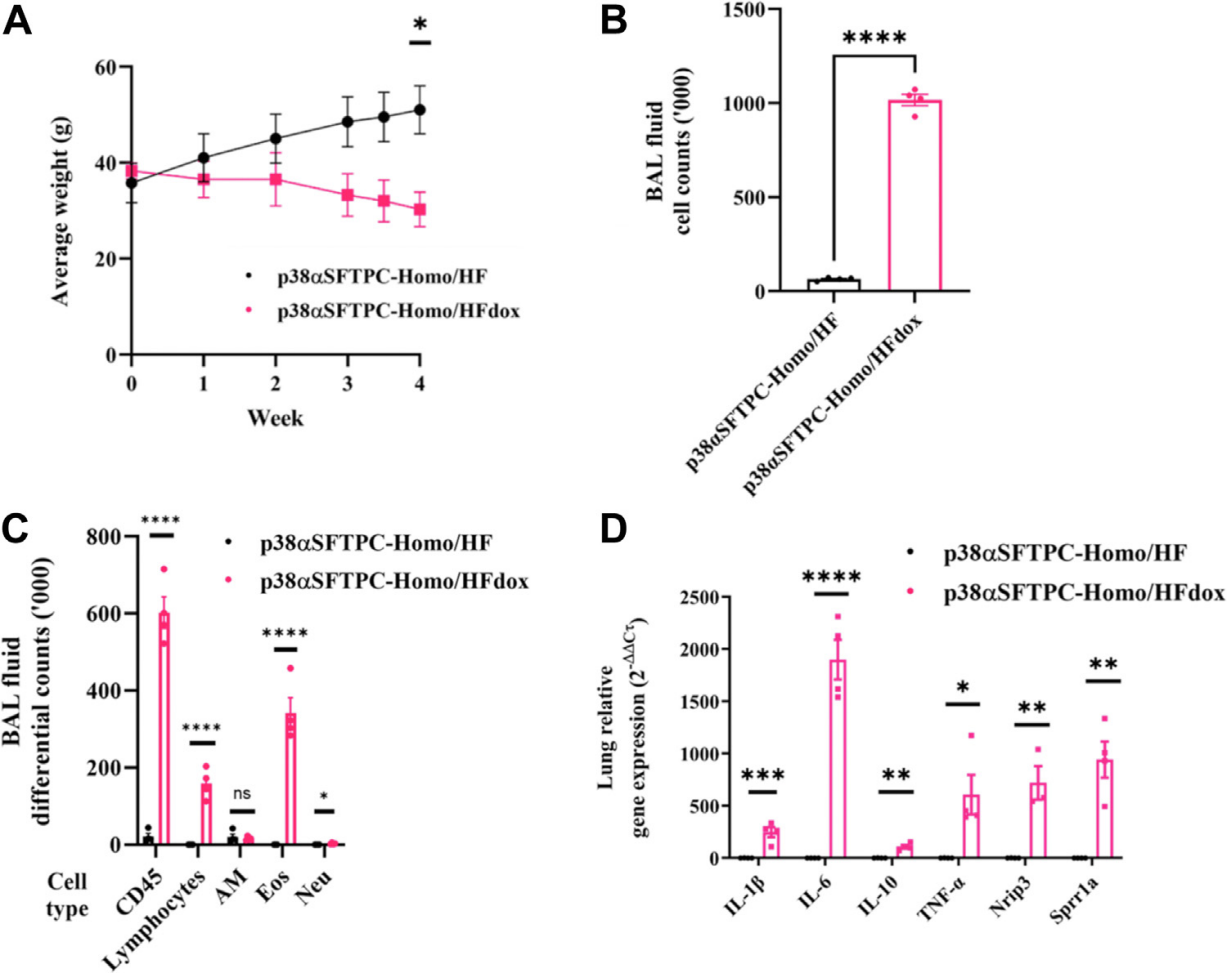
**A combination of AT2-specific p38α^D176A+F327S^ expression and HFD-induced symptoms of airway inflammation in male p38αSFTPC-homo mice.***A*, body weights of male p38αSFTPC-homo mice provided with either HF-Dox–supplemented diet or HFD for 4 weeks. *B*, counts of cells obtained from BAL fluid of p38αSFTPC-homo mice provided with the HF-Dox–supplemented diet or HFD. *C*, differential counts for individual cell subpopulations from BAL fluid of p38αSFTPC-homo mice provided with the HF-Dox–supplemented diet or HFD. *D*, relative mRNA levels encoding the indicated proteins in lungs of p38αSFTPC-homo mice provided with the HF-Dox–supplemented diet or HFD. *A*–*D*, (n = 4 mice). AT2, alveolar type 2; BAL, bronchoalveolar lavage; Dox, doxycycline; HDM, house dust mite; HFD, high-fat diet.

The epidemic of obesity caused a dramatic increase in the rate and severity of respiratory diseases such as asthma (38, 39, 40, 41, 42, 43). Recent observations point to p38α as a potential mediator of this association (44, 45, 46, 47, 48; See Discussion). Therefore, we tested whether the combining p38α^D176A+F327S^ expression with an HFD in the p38αSFTPC mouse model would affect the lungs. We observed that while male p38αSFTPC-homo mice provided with HFD consistently gained weight, the same mice provided with high-fat (HF)-Dox–supplemented diet (namely, expressing p38α^D176A+F327S^ and fed with HFD) began to lose weight 3 to 4 weeks after initiating the experiment (Fig. 6*A*). Significant cellular infiltration was detected in BAL fluid from these mice (Fig. 6*B*) and FACS analyses revealed them to be mostly eosinophils and lymphocytes (Fig. 6*C*). On the other hand, p38αSFTPC-homo mice that had been provided with HFD alone presented no significant airway infiltration.

Searching for the potential mechanism by which p38α^D176A+F327S^ could impose these inflammatory effects, we monitored levels of cytokines and chemokines in the lungs. Relative expression levels of mRNAs encoding inflammatory cytokines including interleukins (IL-1β, IL-6, and IL-10) and tumor necrosis factor-α (TNF-α) were upregulated in the lungs of p38αSFTPC-homo mice given HF-Dox–supplemented diet compared to those in mice fed with HFD only (Fig. 6*D*). Notably, IL-1β, IL-6, and TNF-α are major proinflammatory cytokines known to be involved in obesity-induced inflammation (49). Relative expression levels for some genes seen to be consistently upregulated in our RNA-seq analyses such as *Nrip3* and *Sprr1a* were also measured, and RT-PCR confirmed that these genes were upregulated in the lungs of p38αSFTPC-homo provided with a HF-Dox–supplemented diet as well (Fig. 6*D*).

**Figure 7:**
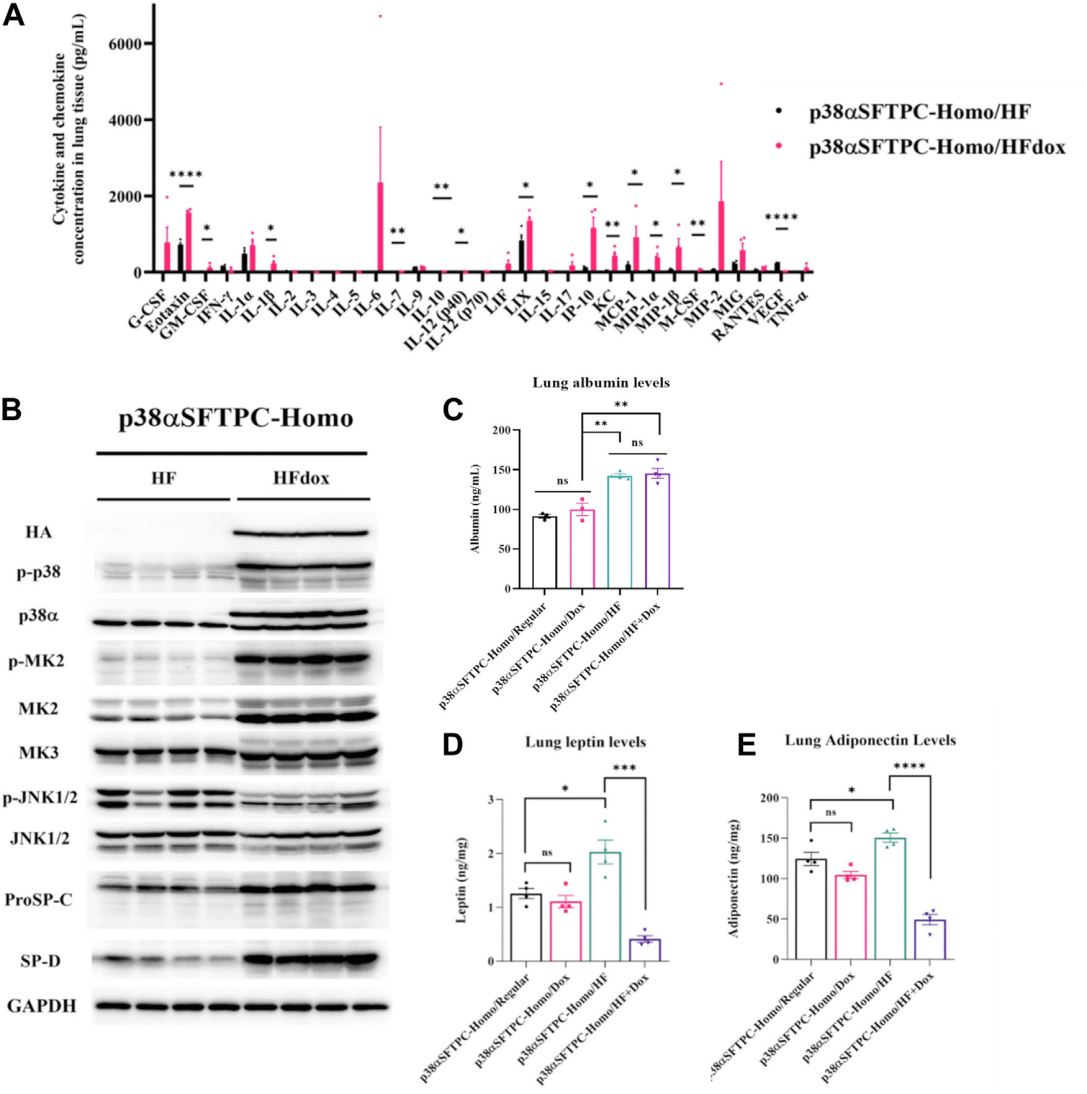
**HFD and AT2-specific p38α^D176A+F327S^ induce differing outcomes in male p38αSFTPC-homo mice by themselves or in combination.***A*, concentrations of the indicated cytokines and chemokine in whole lung lysates prepared from p38αSFTPC-homo mice provided with a HF-Dox–supplemented diet or HFD. *B*, Western blot analysis of whole lung lysates prepared from p38αSFTPC-homo mice provided with HF-dox–supplemented diet or HFD for 4 weeks *C*, albumin levels were measured in whole lung lysates prepared from mice provided with regular, Dox-supplemented, HF, or HF-Dox–supplemented diet for either 4 weeks or 3 months. *D*, levels of leptin was measured in whole lung lysates prepared from mice provided with regular, Dox-supplemented, HF, or HF-Dox–supplemented diet for either 4 weeks or 3 months. *E*, levels of adiponectin was measured in whole lung lysates prepared from mice provided with regular, Dox-supplemented, HF, or HF-Dox–supplemented diet for either 4 weeks or 3 months. Representative blots are shown. *A*–*E*, (n = 3–4 mice). AT2, alveolar type 2; Dox, doxycycline; HFD, high-fat diet.

A Luminex assay further revealed upregulation of cytokines such as IL-1β as well as chemokines involved in eosinophil and lymphocyte chemotaxis including eotaxin, interferon-gamma-induced protein 10, macrophage inflammatory protein 1α, and macrophage inflammatory protein 1β at the protein level. Interestingly, vascular epithelial growth factor was the only molecule significantly reduced in male p38αSFTPC-homo mice fed with HF-Dox–supplemented diet (Fig. 7*A*). These observations suggest that the upregulation of such cytokines and chemokines in the lungs is at least partially responsible for the observed eosinophil and lymphocyte infiltration within the airway. Elevated levels and phosphorylation of the proinflammatory kinase MK2 as well as proSP-C and SP-D in mice provided with a HF-Dox–supplemented diet (Fig. 7*B*) are likely also related to the underlying pathological mechanism. Interestingly, the level of phosphorylated c-Jun-N-terminal kinase 1/2 (JNK1/2) was lower in mice provided with HF-Dox–supplemented diet than in those fed with HFD alone (Fig. 7*B*).

Female p38αSFTPC-homo mice fed with HF-Dox–supplemented diet were also affected but all pathological outcomes were less robust when compared to male mice (Fig. S3). For example, female p38αSFTPC-homo mice did not exhibit weight loss when provided with a HF-Dox–supplemented diet (Fig. S3*A*), and their BAL fluid cell counts (∼100 k on average) were lower when than those measured in male mice (∼1 million on average) (Fig. S3*B*). Nonetheless, eosinophils remain the major infiltrating immune cell population within the airway of female mice (Fig. S3*C*). Furthermore, changes in expression levels of phospho-MK2, phospho-JNK1/2, and SP-D in female mice (Fig. S3*D*) were similar to those seen in male mice. This difference between male and female mice may be related to a known phenomenon in human males, which are likelier to develop eosinophilic asthma (50).

### Lung albumin levels are increased in mice fed with an HFD regardless of p38**α**^D176A+F327S^ expression

We also tested lung albumin levels in p38αSFTPC-homo mice fed with various diets for periods of 4 weeks to 3 months. Interestingly, lung albumin levels were significantly upregulated in p38αSFTPC-homo mice fed with HFD or HF-Dox–supplemented diet for 4 weeks as compared to p38αSFTPC-homo mice fed with a regular or Dox-supplemented diet for 3 months (Fig. 7*C*). No significant differences in lung albumin levels were observed between mice fed with a regular or Dox-supplemented diet for 3 months or between mice fed with HFD or HF-Dox–supplemented diet for 4 weeks. This suggests that while HFD alone can induce some degree of damage to the lung, the differing observations between mice fed with HFD or HF-Dox–supplemented diet are mediated by other mechanisms.

### Leptin and adiponectin are decreased in the lungs of mice expressing p38**α**^D176A+F327S^ and fed with an HFD

To reveal the underlying mechanism behind the lack of weight gain in p38αSFTPC-homo mice fed with a HF-Dox–supplemented diet, we monitored the levels of leptin and adiponectin, critical adipokines suggested to play opposing roles in obesity and its associated conditions. In obesity-associated lung conditions, leptin is believed to be proinflammatory while adiponectin is anti-inflammatory in nature (51). We tested the levels of both adipokines in the lungs of male p38αSFTPC-homo mice fed with various diets for a period of 4 weeks to 3 months (Fig. 7*D*). In p38αSFTPC-homo mice fed with a regular or Dox-supplemented diet for 3 months, no significant differences were noted in the levels of leptin or adiponectin in the lung. When p38αSFTPC-homo mice were fed with HFD for 4 weeks, a significant increase in the levels of leptin and adiponectin was noted. Yet, in p38αSFTPC-homo mice fed with a HF-Dox–supplemented diet for 4 weeks, levels of leptin and adiponectin in the lung were markedly decreased, dropping even lower than in mice fed with a regular diet (Fig. 7*D*). Thus, chronic activation of p38α suppresses the HFD-induced elevation of leptin and adiponectin. The exact roles of both adipokines in the lung are not fully clear (51). Yet, adiponectin is reported to be anti-inflammatory in nature, and its downregulation in the lungs of p38αSFTPC-homo mice given a HF-Dox–supplemented diet could explain the inflammatory outcomes observed.

Taken together, mice subjected to the combination of HFD and chronic p38α activation in the lung exhibited signs of ongoing inflammation, characterized by eosinophil and lymphocyte infiltration within the airways. These mice also fail to exhibit weight gain, and their inflammatory symptoms seem to be governed by increased levels of proinflammatory cytokines and chemokines such as IL-1β and IL-6, TNF-α, and eotaxin, as well as the activation of proinflammatory kinases such as MK2. A decrease in adipokines such as adiponectin may also contribute to the observed inflammatory symptoms and the lack of weight gain in response to HFD.

## Discussion

This study presents the first transgenic mouse model capable of lung-specific p38α activation. The model takes advantage of the development of intrinsically active p38α variants that are independent of upstream regulation (25, 26) and of the establishment of a double-cassette expression system in mice (27). To achieve lung-specific expression with our approach, a custom-built transgenic mouse line which constitutively expresses Cre in AT2 pneumocytes was generated. Commercially available mouse strains harboring expression systems of *Sftpc* promoter-driven Cre were unsuitable because they require the use of other inducers such as tamoxifen for Cre expression (Sftpc-CreER^T2^) or bypass Cre by constitutively expressing rtTA instead under the *Sftpc* gene promoter (Sp-c-rtTA) (30). As such, we generated SFTPC-Cre mice to ensure tight regulation over the temporal and spatial aspects of transgene expression by necessitating the presence of both Cre and Dox.

The observation that expression of the HA-tag is restricted to cells expressing the AT2-specific marker ProSP-C further affirms that our system allows for precise control over p38α^D176A+F327S^ expression. Our findings demonstrate that p38α^D176A+F327S^ is expressed only when Dox is provided, and this is highly specific to AT2 cells in p38αSFTPC mice.

Given the effect of p38α^D176A+F327S^ on other tissues (27, 28; Gilad *et al.*, unpublished data), we expected that its expression *per se* would evoke pathological symptoms in the lung as well. However, chronic expression of p38α^D176A+F327S^ was insufficient to induce or exacerbate lung pathology. Furthermore, p38α^D176A+F327S^ expression in the liver and muscle previously led to the downregulation of MK2 and MKK6. In those tissues, perhaps the pathological effects of p38α are consequences of downregulation (rather than of upregulation) of downstream components (52). The lack of any phenotypic or biochemical effect from p38α^D176A+F327S^ is difficult to explain. Perhaps p38α activation in other lung cells, rather than AT2 pneumocytes, is a driver of chronic respiratory diseases. Also, RNA-seq analyses and monitored protein expression levels were carried out on whole lung extracts, raising the possibility that the outcomes of p38α^D176A+F327S^ expression in AT2 cells are “diluted” by other cell types in the lung. In the human lung, AT2 cells comprise just 15% of all cells (53). This contrasts with the liver and muscle, which are generally more homogeneous in nature (consisting largely of hepatocytes and myoblasts, respectively).

A common observation between p38αSFTPC-homo mice and our prior model expressing p38α^D176A+F327S^ across all mouse tissues was the upregulation of Arg1^+^ M2-like AMϕ in the lungs (28). Furthermore, when mice expressing p38α^D176A+F327S^ were challenged with HDM extract, which is known to increase M2-like AMϕ numbers (37), this upregulation was amplified. This suggests that increased p38α activity in AT2 cells could be somehow inducing the polarization of AMϕ toward an M2-like phenotype.

Apart from maintaining immune homeostasis, other key AT2 functions include lung surfactant production and serving as progenitor cells of the alveolar epithelium (12). Perhaps the nature of p38α activity in alveolar homeostasis is regulating the production of surfactants such as surfactant protein A (SP-A) and SP-D which are largely involved in innate immunity and host defence against pulmonary infections (54). This notion might also explain the upregulation of Arg1^+^ M2-like AMϕ, since surfactants such as SP-A are reportedly capable of inducing M2-like polarization (55). As mentioned earlier, increased expression of SP-D is associated with diseases such as COPD, suggesting that this might be one outcome from the increased levels of p38α activity seen during such conditions.

Most importantly, we demonstrate that p38α could potentially be a critical link between obesity and its associated respiratory conditions. Obese individuals are prone to developing more severe chronic respiratory disease outcomes (8, 56), and the prevalence of obese individuals with concomitant lung diseases is increasing (38, 39, 40, 41, 42, 43). Animal models also support the linkage between obesity and the severity of associated respiratory diseases (17, 44, 45, 46). Studies involving isolated primary cells from obese patients further confirm that obesity affects pathological outcomes of respiratory conditions (57, 58). The notion that elevated p38α activity could function as the bridge (or even be responsible for) between obesity and respiratory pathology is also emerging. This is evident in studies involving the use of p38α inhibitors in animal models (44, 45, 46), as well as exposure of isolated primary cells to fatty acids (47). The observations in p38αSFTPC mice, in which HFD alone is insufficient to promote any obesity-associated respiratory disease, could explain why only some, but not all obese individuals will develop such conditions (17). Hypothetically, it might be possible that in some individuals, factors such as stress, genetic polymorphisms, airborne pollution, or allergies could induce chronically elevated p38α activity, making them more prone to developing HFD-mediated lung diseases. In contrast, obese individuals who do not possess increased levels of p38α activity may not develop associated lung conditions as readily.

The mechanism by which p38α^D176a+F327S^, when combined with HFD, causes lung pathology and prevents weight gain primarily involves the induction of proinflammatory cytokines such as IL-1β, IL-6, and TNF-α, probably via activation of MK2, a known mediator of p38α-driven inflammatory signaling. While HFD alone appears to cause some ongoing damage to the lung, as evidenced by an upregulation of lung albumin levels, it appears that other mechanisms are involved, which explains the stronger pathological outcomes in mice exposed to a combination of HFD and AT2-specific p38α^D176A+F327S^ expression.

The combination of HFD and p38α activity also causes a reduction in the levels of leptin and adiponectin within the lung. While leptin is more commonly associated with inflammation, leptin deficiency in mice has also been shown to result in heightened inflammatory responses under certain conditions (59). Perhaps losing the protective functions of leptin and adiponectin in the lung further enhances the elevation of cytokines and appearance of inflammatory markers.

The difference in response to HFD between male and female mice is not entirely unexpected, since gender is known to impact obesity and is an important factor when modeling obesity-associated respiratory diseases (60, 61). Sex hormones such as oestrogen are suggested as possible factors for these observations, with notable gender-specific differences in conditions such as asthma, COPD, and IPF reported across animal models and human patients (62).

The combination of p38α activity and HFD causes some downregulation of JNK1/2 level and activity, showing that crosstalk between these stress kinases, reported in other systems (63, 64), exists in the lung. It is not known at this point whether downregulation of JNK1/2 is associated with induction of inflammatory symptoms.

Notably, we reported here the first transgenic mouse line (SFTPC-Cre) to constitutively express Cre under the endogenous *Sftpc* gene promoter as well as the first transgenic mouse model (p38αSFTPC) for the controllable lung-specific activation of p38α. These models can now be applied in various studies of the lung, particularly to understand the role of p38α within this tissue. The observed symptoms of lung inflammation in mice exhibiting AT2-specific elevated p38α activity and fed with HFD suggests the use of the model for deciphering the basis of obesity-related lung diseases. This includes asthma, which is frequently characterized by eosinophilia and lymphocyte infiltration into the lungs. Our findings reveal that p38α activity alone is insufficient for the development of inflammatory outcomes. However, it suggests a potential explanation for the predisposition of some obese individuals, that is, those with elevated p38α activity, toward severe outcomes in HFD-mediated respiratory diseases.

## Experimental procedures

### Antibodies and reagents

Primary antibodies used for immunoblotting and immunofluorescent staining were as follows: HA-tag (6E2) mouse mAb, p-p38, p38α, MK2, MK3, pMK2, p-MKK3/6, MKK3, MKK6, MAPK phosphatase-1 (all rabbit) (Cell Signaling Technologies); horseradish peroxidase conjugated GAPDH and β-actin (Proteintech Group); anti-HA high affinity from rat IgG1 (Roche); ProSP-C (Merck Millipore); SP-ASP-D (Abcam). Secondary antibodies used are as follows: anti-rabbit, anti-rat, anti-mouse (Cell Signaling Technologies), Alexa Fluor 488–conjugated anti-rabbit, Alexa Fluor 555–conjugated anti-mouse (Thermo Fisher Scientific).

Fluorochrome-labeled antibodies used for flow cytometry: Pe/Cy7 anti-mouse CD45, PE anti-mouse CD170 (Siglec F), APC anti-mouse CD11b, PerCp-Cy5.5 anti-mouse Gr1, APC/Cy7 anti-mouse NOS2 inducible nitric oxide synthase, APC anti-mouse CD206, PerCp-eFluor 710 anti-mouse CD80 (Thermo Fisher Scientific), FITC anti-mouse Arg-1 (R&D Systems), and PB anti-mouse CD11c (BioLegend).

### Transgenic mouse strains

Transgenic mice expressing Cre recombinase under the *Sftpc* promoter (SFTPC-Cre) were generated at the Animal Gene Editing laboratory, Institute of Molecular and Cell Biology, A∗STAR. The mice were first crossed with C57BL/6Jinv (Jackson Laboratories) mice purchased from InVivos, for two generations to make their genetic background closer to that of C57BL/6J. F_3_ generation mice were then bred with “carrier” mice harboring the p38α^D176A+F327S^ inducible expression system in the Rosa26 locus, generating “p38αSFTPC-hetero” mice. These were further inbred to obtain “p38αSFTPC-homo” mice.

Mice were maintained in a 12 h light-dark cycle with food and water available *ad libitum*. Cre^+^/p38α^D176A+F327S^ mice were provided with a Dox-supplemented diet (625 mg/kg) (TD.01306) (Envigo). For HFD and HF+dox assays, Cre^+^/p38α^D176A+F327S +/+^ mice were provided with an adjusted calories diet (42% from fat) (TD.88137) or a Dox-supplemented (625 mg/kg) 21% anhydrous milk fat diet (TD.180724), respectively.

Mice were sacrificed via anesthesia overdose using an i.p. injection of a ketamine/medetomidine mixture to minimise pain and distress. This process was followed by cardiac puncture and exsanguination. Animal experiments were performed according to the guidelines laid out and approved by the International Animal Care and Use Committee of the National University of Singapore (Animal protocol numbers: BR20-0795, R20-0799).

### Animal disease models

p38αSFTPC-homo mice were first provided with a Dox-supplemented or regular diet for 2 weeks prior to being subjected with one of the following disease models (1). LPS-induced acute lung inflammation model, 10 mg/kg of LPS from *Escherichia coli* O111:B4 (10 mg/kg) (Sigma-Aldrich) in 40 μl of normal saline was delivered intratracheally. Mice were sacrificed 24 h later for sample collection (2). HDM-induced allergic asthma model, 50 μg of *Dermatophagoides pteronyssinus* HDM extract (Greer Laboratories) in 40 μl of normal saline was delivered intratracheally on days 0, 7, and 14. Mice were sacrificed 3 days after the third HDM dose on day 17 for sample collection (3). HFD model—p38αSFTPC mice were provided with either a regular diet, Dox-supplemented diet, HFD, or Dox-supplemented HFD(Envigo) as described in “Results.” Mice were sacrificed after 4 to 6 weeks depending on the assay.

### Polymerase chain reactions

#### PCR for mice genotyping

Tail clippings were obtained from bred transgenic mice at 3 weeks old. Samples were stored at −20 °C for no more than a week before DNA extraction. Genomic DNA was extracted using a REDExtract-N-Amp Tissue PCR kit (Sigma-Aldrich) according to the manufacturer’s protocol. PCR was carried out and PCR products separated on a 2% agarose gel. Primers used are listed in Table S1*A*.

#### Quantitative real-time PCR for gene expression measurements

cDNA was synthesized using an iScript gDNA Clear cDNA Synthesis Kit (Bio-Rad) according to the manufacturer’s instructions. One milligram of RNA was used for each reaction. Quantitative real-time PCR was conducted using a 7500 Fast Real-Time PCR (Model 4351106, Applied Biosystems) with iTaq Universal SYBR Green Supermix (Bio-Rad). Results are presented as 2^-ΔΔCt^ normalized against the 18S ribosome fragment as a housekeeping gene. The genes measured and their respective primers are listed in Table S1*B*.

### RNA-seq analysis

Detection of differentially expressed genes—raw reads were quality-trimmed and remaining adapter sequences removed using cutadapt. Processed reads were aligned to the mouse genome version GRCm39 (genome annotations from Ensembl release 106) with TopHat. Quantification was done with htseq-count with strand information set to “reverse.” DESeq2 (obtained from https://bioconductor.org/packages/release/bioc/html/DESeq2.html) analysis for identification of differentially expressed genes was performed and pairwise comparisons tested with default parameters but not using the independent filtering algorithm. The significance threshold was set to *p* adj < 0.1. In addition, significant genes were further filtered by their log2 fold change value. This filtering was baseMean-dependent and required a baseMean >5 and an absolute log2 fold change higher than 5/sqrt(baseMean) + 0.5. This ensures that while highly expressed genes require a fold change of at least 1.5 in order to pass the filter, it is more stringent for genes with low expression, which now require a fold change of at least 7 to pass the filter. Datasets are included in Supporting Information as Datasets S1 and S2.

### Immunoblotting

Lysates were first prepared from mouse tissue and cell samples using a lysis buffer consisting of T-Per Tissue Protein Extraction reagent and Halt Protease and Phosphatase Inhibitor Cocktail (Thermo Fisher Scientific). Tissues were lysed in a bullet blender with 500 to 700 μl of lysis buffer before total protein was extracted, stored in Laemmli buffer, and heated at 100 °C for 10 min prior to storage at −20 °C. Equal amounts of protein per sample were resolved via SDS-PAGE and transferred to nitrocellulose membranes using the Trans-blot Turbo transfer system (Bio-Rad). Membranes were blocked with 5% bovine serum albumin (Sigma-Aldrich) diluted in Tris-buffered saline with 0.1% Tween 20 and later incubated accordingly with the relevant primary antibodies and their appropriate secondary antibodies. Antibodies were diluted in 1% bovine serum albumin in Tris-buffered saline with 0.1% Tween 20. Blots were visualized using a ChemiDOC Touch Imaging System (Bio-Rad) and SuperSignal West Femto Maximum Sensitivity Substrate Kit (Thermo Fisher Scientific). Apparent molecular weights of the detected proteins were compared against the antibody manufacturer’s reports to ensure that they were in the correct position. When blots were quantitatively analyzed, ImageJ software (obtained from https://imagej.net/ij/download.html) was used.

### Bronchoalveolar lavage

Mouse BAL fluid was obtained via insertion of a cannula into the trachea following tracheotomy. Lungs were flushed with three rounds of 0.5 ml ice-cold 1x PBS, and BAL fluid obtained was centrifuged to obtain cell pellets for downstream FACS analyses. Supernatants obtained were stored at −80 °C for further assays.

### Fluorescence-activated cell sorting

Cell pellets were re-suspended in 1x red blood cell lysis buffer (BioLegend) to remove any contaminating erythrocytes before staining with an established antibody panel for identification of relevant immune cell types. Respective single color controls were prepared with compensation beads according to the manufacturer’s protocol. For intracellular staining, an additional fixation and permeabilization step was carried out using an intracellular fixation and permeabilization kit (Thermo Fisher Scientific) according to the manufacturer’s protocol. Flow cytometric analyses were carried out using either a BD LSRFortessa Cell Analyzer or a BD LSRFortessa X-20 Cell Analyzer (BD Biosciences, Franklin Lakes) flow cytometer.

FACS data and .fcs files were analyzed using FlowJo software (obtained from https://www.flowjo.com/download) (FlowJo LLC). Further statistical analysis was carried out using GraphPad Prism software (obtained from https://www.graphpad.com/) (GraphPad Software).

### Histological examination

Whole mouse lungs were perfused with 0.5 ml of 10% neutral-buffered formalin (Thermo Fisher Scientific) via an intratracheal cannula following sacrifice. The trachea is then secured with a surgical suture to prevent leakage while it is transferred and immersed in 5 ml NBFneutral-buffered formalin for 48 h. Whole lung tissues were then transferred to 70% EtOH. Tissue processing, paraffin embedding, as well as sectioning and slide fixation was carried out at the Advanced Molecular Pathology Laboratory, A∗Star. Lung tissue sections were later prepared accordingly and stained with fluorescent antibodies. Antigen retrieval was carried out using a Tris-EDTA buffer, pH 9, in a 2100 Antigen Retriever (Aptum Biologics). H&E staining was carried out according to the manufacturer’s protocol (Sigma-Aldrich).

Slides were imaged at 100 to 400x magnifications. Further image analysis was carried out using ImageJ software (National Institutes of Health).

### Luminex assay

A MILLIPLEX MAP Mouse Cytokine/Chemokine 25-plex Magnetic Bead Panel (Merck Millipore) was carried out on a Luminex MAGPIX system (Thermo Fisher Scientific) according to the manufacturer’s protocol. BAL fluid samples were obtained as described in “Bronchoalveolar lavage.” Lung lysates were prepared as described in “Immunoblotting.”

### Enzyme-linked immunosorbent assay

ELISA kits for mouse leptin (Proteintech Group), adiponectin (R&D Systems), and albumin (Abcam) were carried out according to manufacturer’s instructions. Lung lysates were prepared as described in “Immunoblotting,” with the only difference being that 1x PBS with Halt Protease and Phosphatase Inhibitor Cocktail was used as lysis buffer.

### Statistical analysis

The data in this manuscript is presented as mean ± SEM. Statistical significance of differences between groups was calculated using the two-tailed unpaired *t* test with GraphPad Prism software (∗*p* < 0.05, ∗∗*p* < 0.01, ∗∗∗*p* < 0.001, and ∗∗∗∗*p* < 0.0001).

## Data availability

All data described can be found within this article.

## Supporting information

This article contains supporting information.

## Conflict of interest

D. E. holds a Wolfson family chair in Biochemistry. The other authors declare that they have no conflicts of interest with the contents of this article.

## Supporting information

Supporting information

RNASeq Dataset S1

RNASeq Dataset S2
