# Supplementary material for: A combination of alveolar type 2–specific p38α activation with a high-fat diet increases inflammatory markers in mouse lungs
Source: J Biol Chem. 2025 Mar 19;301(4):108425. doi: 10.1016/j.jbc.2025.108425 (PMC12018981; doi:10.1016/j.jbc.2025.108425)
Supplement: Supporting information [file mmc1.pdf]

A combination of alveolar type 2-specific p38α activation with a high-fat diet increases inflammatory markers in mouse lungs

C. K. Matthew Heng[a, b], Ilona Darlyuk-Saadon[a,b], Liao Wupeng[b,c], Manju P. Mohanam[a,b], Phyllis X. L. Gan[b,c], Nechama Gilad[b,d], Christabel C.M.Y. Chan[c,e], Inbar Plaschkes[f], W. S. Fred Wong[b, c, e,*], David Engelberg[a,b,d,*]

Contents

Table S1A. A list of primer sequences for mouse genotyping.

Table S1B. A list of primer sequences for RT-PCR analysis.

Table S2. Genes used to define alveolar epithelial cell subsets.

Figure S1. A schematic representation of the transgene cassette used for inducible expression of p38α$^{D176A+F327S}$ in a spatially and temporally controllable manner.

Figure S2. Expression of p38α$^{D176A+F327S}$ had minimal effect on the lung mRNA repertoire.

Figure S3. Female p38αSFTPC-Homo mice expressing p38α$^{D176A+F327S}$ exhibit a markedly less robust phenotype after being fed with a dox-supplemented-HF diet.

Dataset S1-2. Bulk RNA-Seq datasets of DEGs from p38αSFTPC-Homo and WT mice provided with the appropriate dox-supplemented or regular diet.

PCR product	Forward 5’-3’	Reverse 5’-3’	Product size
p38$\alpha^{D176A+F327S}$ 5’End	TCCAGCCCGACCTCCCCTGGCACAACG	GGCATTAAAGCAGCGTATCC	292
p38$\alpha^{D176A+F327S}$ 3’End	ATTGGGAAGACAATAGCAGGCATGC	TCAAAGAGCAGCGAGAAGCGTTCAG	229
Cre	CCGGTGAACGTGAAAAACAGGCTTCTA	GATTAACATTCACCCACCGTCAGT	206
R26-211	TTGCCTCAAGAGGGGCGTGCTGAGCCAG	AGGACAACGCCCACACACCAGGTTAGC	378
Sftpc	TGTCAGGGGACCTACTAGGTATCTC	CGTCCTTCGACGAGGACGGTCTTT	225

Table S1A: A list of primer sequences for mouse genotyping.

Gene name	Forward 5’-3’	Reverse 5’-3’
18S Ribosome	GTAACCCGTTGAACCCCATT	CCATCCAATCGGTAGTAGCG
p38α human	GCCGAGCTGTTGACTGGAAG	GGAGGTCCCTGCTTTCAAAGG
IL-1β	GCAACTGTTCCTGAACTCAACT	ATCTTTTGGGGTCCGTCAACT
IL-6	GATGCTACCAAACTGGATATAATC	TGTACTCCAGGTAGCTATG
IL-10	GCTGGACAACATACTGCTAAC	ATTTCCGATAAGGCTTGGCAA
TNF-α	AAGCCTGTAGCCCACGTCGTA	GGCACCACTAGTTGGTTGTCTTTG
Nrip3	GGTGCAGTTCATCCACAAGGAC	GACAGGTTGGTTTCCATGAGGC
Sprr1a	CAAGGCACCTGAGCCCTGCAA	AGGCTCTGGTGCCTTAGGTTGG
Sftpa1	ACCTGGATGAGGAGCTTCAGAC	CTGACTGCCCATTGGTGGAAAAG
Sftpd	AGGTCCAGTTGGACCCAAAGGA	CTGGTTTGCCTTGAGGTCCTATG

Table S1B: A list of primer sequences for RT-PCR analysis.

Cell type	Gene name
**AT1**	*Pdpn*
	Hopx
	Cav1
**AT2**	*Sftpa1*
	Sftpc
	Soat1
	Lpcat1
**DATP**	*Cldn4*
	Krt8
	Cdkn1a
	Ndrg1
	Sprr1a

Supplementary Table 2: Genes used to define alveolar epithelial cell subsets.

Supplementary figure S1

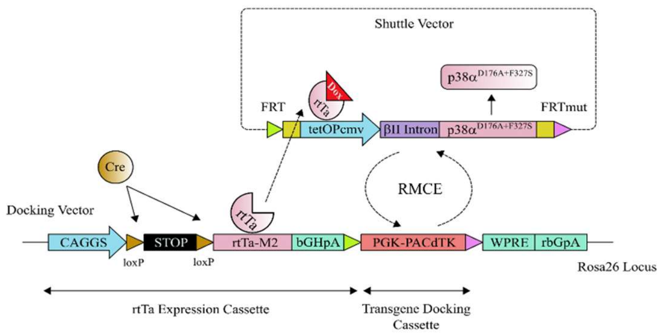

Supplementary figure S1. A schematic representation of the transgene cassette used for inducible expression of p38α$^{D176A+F327S}$ in a spatially and temporally controllable manner. In the first portion of the cassette, rtTa transcription is driven by a constitutively active CAGGS promoter region. However, expression is blocked by a ‘STOP’ sequence flanked by loxP sites. When Cre is present, it excises the ‘STOP’ sequence, thus allowing expression of rtTa. The second portion of the transgene cassette induces expression of p38α$^{D176A+F327S}$ under a TetOn promoter. Thus, both Cre and dox are necessary for transgene expression using this cassette.

Supplementary figure S2

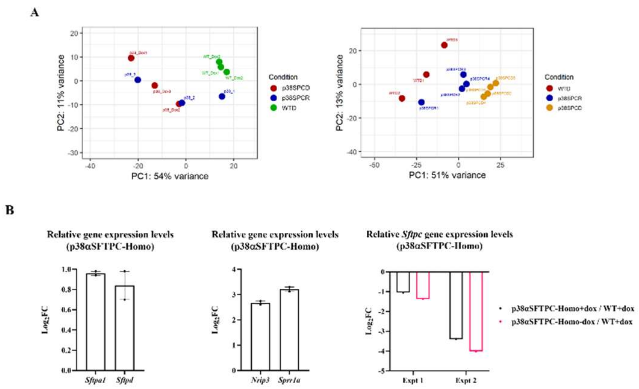

Supplementary figure S2. Expression of p38α$^{D176A+F327S}$ had minimal effect on the lung mRNA repertoire. (A) PCA plots from the first (left panel) and second (right panel) independent RNASeq experiments. 'WTD' refers to WT mice given a dox-supplemented diet, 'p38SPCR' refers to p38αSFTPC-Homo mice given a regular diet, and 'p38SPCD' refers to p38αSFTPC-Homo mice given a dox-supplemented diet. **(B)** Selected $\log_2$ fold changes of: Significantly upregulated genes common between both experiments (left panel); genes encoding for surfactant-associated proteins (middle panel); *Sftpc* gene expression in the lungs of p38αSFTPC-Homo mice fed with a dox-supplemented or regular diet compared to WT mice fed with a dox-supplemented diet (n = 3-4 mice).

Supplementary figure S3

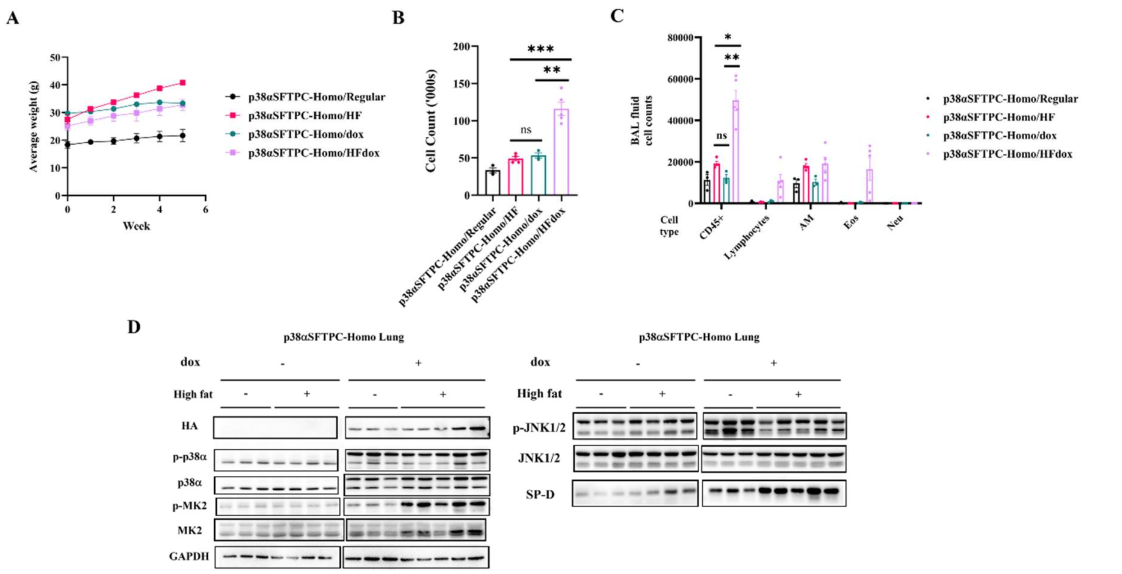

Supplementary figure S3. Female p38αSFTPC-Homo mice expressing p38α$^{D176A+F327S}$ exhibit a markedly less robust phenotype after being fed with a dox-supplemented-HF diet. (A) Average weights of each group of mice over 5 weeks. **(B)** BAL fluid cell counts from female p38αSFTPC-Homo mice after 5 weeks on their respective diets **(C)** Differential BAL fluid cell counts from the same time. **(D)** Western blot analysis of the indicated proteins in the lungs of female p38αSFTPC-Homo mice fed with their respective diets. Representative blots are used. **(A-D)** (n = 3-5 mice).

Dataset S1-2. Bulk RNA-Seq datasets of DEGs from p38αSFTPC-Homo and WT mice provided with the appropriate dox-supplemented or regular diet. Two independent experiments were conducted and their DEG datasets are included here as Dataset S1.xlsx and Dataset S2.xlsx. p38αSFTPC-Homo mice were provided with a dox-supplemented or regular diet for two weeks prior to sacrifice. WT mice provided with a dox-supplemented diet were used as a control for the effect of dox. (n = 3-4 mice).
